# Lessons from PrEP: A Qualitative Study Investigating How Clinical and Policy Experts Weigh Ethics and Evidence When Evaluating Preventive Medications for Use in Pregnant and Breastfeeding Women

**DOI:** 10.1007/s10461-018-2361-5

**Published:** 2018-12-14

**Authors:** Kristin M. Beima-Sofie, Susan Brown Trinidad, Kenneth Ngure, Renee Heffron, Jared M. Baeten, Grace C. John-Stewart, Maureen Kelley

**Affiliations:** 1 Department of Global Health, University of Washington, Seattle, WA 98104 USA; 2 Department of Bioethics and Humanities, University of Washington, Seattle, WA USA; 3 Department of Epidemiology, University of Washington, Seattle, WA USA; 4 Department of Pediatrics, University of Washington, Seattle, WA USA; 5 Department of Medicine, University of Washington, Seattle, WA USA; 6 Department of Community Health, Jomo Kenyatta University of Agriculture and Technology, Nairobi, Kenya; 7 The Ethox Centre and Wellcome Centre for Ethics & Humanities, Nuffield Department of Population Health, University of Oxford, Oxford, England, UK

**Keywords:** Decision-making, Pregnancy and HIV, PrEP, Ethics, Women

## Abstract

This study explored how multinational HIV experts weigh clinical, evidential, and ethical considerations regarding pre-exposure prophylaxis in pregnant/breastfeeding women. Semi-structured interviews were conducted with experts in HIV policy, research, treatment, and implementation from three global regions. A constant comparative approach identified major themes. Experts noted that exclusion of pregnant women from research limits evidence regarding risks/benefits, emphasizing that underinclusion of pregnant women in RCTs shifts the onus of evidence-building to clinical care. Experts discussed approaches for weighing evidence to make decisions, including triangulating evidence from sources other than RCTs. Likelihood and severity of disease strongly influenced decisions. Less effective interventions with limited fetal risk were preferred over interventions of uncertain safety, unless the disease was serious. Experts resisted the dichotomous choice between protecting maternal and fetal interests, arguing that these interests are intertwined and that more holistic approaches to maternal–fetal balance support greater inclusion of pregnant women in research.

**Electronic supplementary material:**

The online version of this article (10.1007/s10461-018-2361-5) contains supplementary material, which is available to authorized users.

## Introduction

The development of safe and effective biomedical interventions for women during pregnancy and breastfeeding is critical. However, historically, pregnant women have been considered a vulnerable population meaning that the ethical threshold for inclusion in research is higher than non-pregnant participants given concerns about potential risks to the developing fetus. This position placed pregnant women in the same category as other dependent populations considered to be more susceptible to harms or exploitation in research, such as children, prisoners, or those suffering from mental illness. Despite recent ethics and policy arguments advocating a shift away from classifying pregnant women as a vulnerable population in research, significant barriers remain to the inclusion of pregnant women in clinical trials given persistent concerns about risks to the fetus [1–4]. In the absence of randomized control trial (RCT)-derived data on safety and efficacy in pregnancy, implementation studies and clinical decisions regarding medications in pregnancy/breastfeeding can be ethically and clinically challenging for health providers, researchers, and policy makers [5–9].

HIV research offers important examples for reflecting on the ethics of inclusion of pregnant women in intervention research. Pregnant women have participated in numerous randomized clinical trials (RCTs) focused on interventions to prevent infant HIV infection. Although the first RCT to evaluate an intervention to prevent mother-to-child HIV transmission (PMTCT), ACTG 076, faced concerns regarding infant safety with antiretroviral drug exposure, in subsequent studies the PMTCT research paradigm shifted away from fears of fetal risk to concern that it may be unethical to withhold zidovudine in placebo-controlled studies of short antiretroviral regimens [10, 11]. Later studies incorporated 3-drug antiretroviral treatment (ART) regimens with potential not only for infant benefits but also for maternal benefits.

Pregnancy is a period of increased risk for HIV acquisition for women in high burden HIV settings [12–14]. Pre-exposure prophylaxis (PrEP) is an effective intervention to prevent HIV infection among women [15, 16]. Although UNAIDS guidelines recommend that pregnant women be included in clinical trials of HIV preventive interventions [17], RCTs involving PrEP discontinued the intervention at detection of pregnancy, limiting data regarding benefit/risk during this period. Despite lack of RCT evidence, WHO guidelines on PrEP use [18], as well as other HIV prevention frameworks [19], promote the use of PrEP by pregnant women in settings of high HIV prevalence.

The case of PrEP during pregnancy offers a valuable opportunity to better understand how clinical, research, and HIV policy experts balance uncertainties and weigh incomplete evidence against ethical considerations when evaluating a new intervention for use by women during pregnancy. Here we report data from a cohort of HIV experts within a larger qualitative study called Choices in Pregnancy (ChIP), which examined the ethical, clinical, and practical considerations in PrEP implementation in pregnancy, including the perspectives of frontline clinicians, women, and partners. Because experts play a critical role in shaping international policy and clinical guidelines on the use of new interventions in pregnancy, it is particularly important to understand how they weigh clinical/evidential, social/cultural, and ethical considerations when considering implementation of interventions and research during pregnancy/breastfeeding. Results from this study can inform the relative weight and value placed on various factors when considering implementation of novel interventions in pregnant women.

## Materials and Methods

### Study Population

Interviews were conducted between February and June 2015. The research team used scientific publications, NGO membership, and other publicly available resources to select a representative sample of informants with professional roles and experiences relevant to the inquiry: policy, clinical, or research expertise in provision of PrEP; clinical care or research in HIV prevention strategies; and/or expertise in research ethics and the inclusion of pregnant women in the context of high HIV disease burden. The team used PubMed literature searches to identify authors from published studies on PrEP, searched national and international databases (CDC, USAID, WHO, NIH) for authors of PrEP guidelines and policies, and used NIH Reporter to identify investigators with ongoing PrEP trials. The team selected 2–4 representatives from key stakeholder groups to recruit. We used snowball sampling to recruit additional participants [20]. We contacted 50 participants via telephone or e-mail to invite their participation and had an enrolment rate of 50%. The rate of decline did not differ between the different expert groups and those who declined often referred colleagues they felt were more qualified to comment on PrEP use in pregnancy. Recruitment was halted when thematic saturation had been reached and participants’ suggestions for additional informants became repetitive [21].

This study was reviewed by the University of Washington IRB and received an exempt determination. All participants provided oral informed consent.

### Data Collection

To ensure consistency and coverage across interviews, our guides included three domains: (1) clinical decision-making during pregnancy/breastfeeding when the effects of a given medication on the developing fetus/nursing infant are uncertain; (2) the ethical conduct of intervention research with pregnant and breastfeeding women; and (3) the clinical implementation of PrEP during pregnancy and breastfeeding in low-resource settings. Interview questions explored decision-making practices, attitudes, and beliefs about medication use during pregnancy using general and PrEP-specific examples. The interview guide was pilot-tested and revised accordingly. (See supplemental material.)

Twenty-five semi-structured interviews were conducted in English by Skype or telephone and ranged between 30 and 60 minutes in length. Interviews were digitally recorded and transcribed. Interviewers prepared and circulated field notes following each interview. Transcripts were verified against audio recordings prior to analysis.

### Data Analysis

We performed a qualitative descriptive analysis, using the constant comparison approach, to develop an initial codebook comprising both deductive and inductive codes (i.e., codes that were derived from the interview questions and codes that captured concepts and themes that emerged from the dataset) [22, 23]. The draft codebook was revised by KBS, SBT, and MK over several iterations. Transcripts were coded using ATLAS.ti v.7 (Scientific Software Development GmbH, Berlin, Germany). KBS and SBT conducted 2 rounds of independent test coding, meeting throughout to reach consensus about the application of specific codes and revisions to the codebook. Once the codebook was finalized, each performed independent coding of half the transcripts, meeting to resolve disagreements and update coding rules, then exchanged transcripts for review of coding application. MK reviewed all transcripts and performed secondary coding on 8 transcripts, selected to represent a range of views, professional roles, and WHO regions.

## Results

The study population included clinicians, ethicists, members of international non-governmental organizations (including WHO, NIH, and CDC), Institutional Review Board/Ethics Review Committee (IRB/ERC) members, and researchers. Participants resided in 3 of the 6 WHO regions: region of the Americas, European region, and African region. Participants self-identified as a mixture of clinicians/healthcare workers, researchers and ethicists, and some (14%) identified as experts from multiple domains. Sixty-eight percent of participants were female; participants reported having between 5 and 45 years’ experience considering issues related to HIV and/or pregnancy (Table 1).

**Table 1:** Participant demographics

Characteristic	n (%) or median (IQR)
Age (years)	48 (41–57)
Female	17 (68%)
WHO region	
Region of the Americas	11 (44%)
African region	12 (48%)
European region	2 (8%)
Self-identified area of expertise^a^	
Clinician/healthcare worker	16
Researcher	15
Ethicist	4
Policy developer	1
Recruitment category	
HIV treatment/prevention in women	12 (48%)
HIV treatment/prevention pediatrics	3 (12%)
HIV policy	5 (20%)
PrEP investigator	5 (20%)
Experience in HIV/MCH (years)	12 (10–23)

^a^Participants could identify more than 1 category

**Fig. 1:**
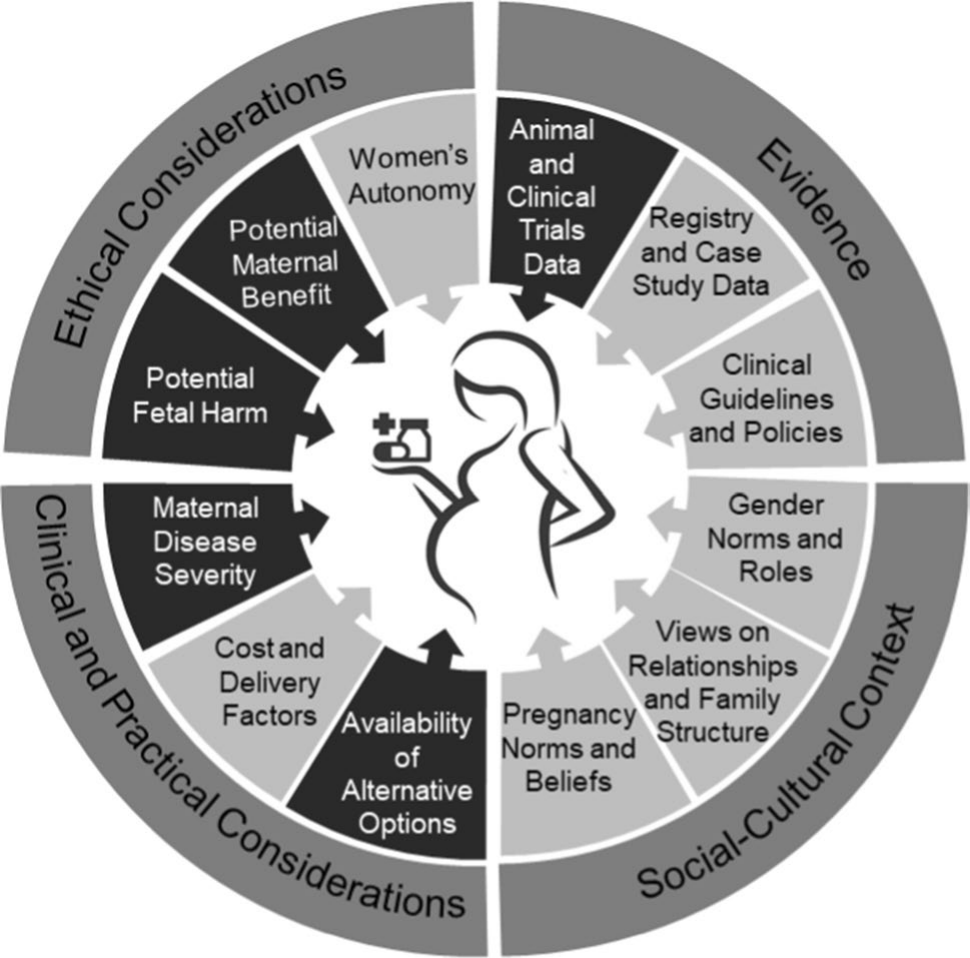
Ethical, evidentiary, practical, and social-cultural considerations that factored in expert decision-making in the provision of medications to pregnant women. Darker emphasis reflects priority given to certain considerations on balance by experts who participated in the study

Our results reveal the complex balancing act faced by clinical and scientific experts when asked to evaluate interventions for use during pregnancy. Relying on PrEP as a case study, experts discussed how they weigh multiple sources of incomplete evidence, ethical concerns, clinical realities, and different social and cultural attitudes about pregnancy and pregnant women. (Figure 1). To describe how experts reason through and weigh these considerations around new interventions for use in pregnancy, study findings are organized by four key themes: (1) developing evidence-based therapies for pregnant women, (2) triangulation of evidence for evaluating treatments in pregnancy, (3) a nuanced approach needed when balancing maternal-fetal risk and benefit, and (4) considering economic factors, logistical constraints, and cultural attitudes about women’s autonomy in local contexts.

### Developing Evidence-Based Therapies for Pregnant Women

All experts in our study noted that designation of pregnant women as a vulnerable group raises the bar for inclusion in research, particularly clinical trials. They described this as a paradox of needing evidence to gather evidence: more evidence-based interventions in pregnancy are needed, but prior evidence is needed to justify inclusion of pregnant women in research. Many experts described how the overall lack of clinical trials data on effectiveness and safety of medications during pregnancy/breastfeeding presents a significant challenge for clinicians and translational scientists. Experts described being caught in a true dilemma: on the one hand, it is difficult to justify the inclusion of pregnant women in clinical research because an intervention is not yet proven safe. On the other, clinicians are making clinical recommendations for pregnant women every day, often without robust evidence, and in the participants’ views, potentially placing women and pregnancies at risk. Participants with a clinical background noted that avoiding risk in the research setting can result in the delivery of substandard clinical care.

“I really do appreciate the sentiment of wanting to be careful, but I think it ends up being that because of this lag, we end up treating women inadequately and inappropriately for periods of time while we wait for data to appear.” **Participant 716; healthcare provider, region of the Americas**

Experts reported that strict exclusion of pregnant women from upstream research displaces risks downstream to clinical care. As they explained, restricting research because of concerns for the safety of the unborn child limits availability of safety data, which ultimately constrains clinicians’ ability to provide evidence-based therapies for pregnant women.

“I think too much caution means that people are just doing stuff on their own when it could lead to more potential harm than doing it in at least a study setting when you [are] gathering more data. I think we need more information, and research is the way to get it.” **Participant 586; healthcare worker/researcher, region of the Americas**

Experts expressed concern that the decision to move to implementation of new interventions in pregnant women is often based on studies with non-pregnant women, leading to untested assumptions about safety during pregnancy or continuation of possibly harmful pre-pregnancy medications.

“I’m on listservs where all these smart people start using whatever great-looking antiretroviral combination is working so well when the woman is not pregnant, and they just continue it during pregnancy. That’s crazy.… They should change to something that they know is safer or safe until there’s enough data to show safety and efficacy in the pregnant woman.” **Participant 408; healthcare worker, region of the Americas**

Several experts noted that medical practice and research remain haunted by past experiences with under-studied drugs, such as thalidomide, which caused serious fetal harm. The resulting fear of fetal harm was thought to be the reason for imposing a high barrier to investigating therapies for pregnant women, inadvertently creating evidence gaps for preventing or treating illness during pregnancy.

“We are so afraid [of fetal risk] that we don’t make a way to investigate this important time…. Because we have not investigated that time of a woman’s life…we’ll have interventions for babies, for adolescents, for non-pregnant women, but the pregnant women will always remain uninvestigated.” **Participant 978; researcher, African region**

When asked about the reasons that might justify greater inclusion of pregnant women in research, most participants thought that investigating treatments for severe conditions affecting women during pregnancy, including HIV, should be given priority. When probed, those participants believed it unethical to exclude pregnant women from research studies when the condition was severe and the interventions could provide direct benefit to the pregnant woman.

“[I]f it’s a drug that might be needed to treat a condition in a woman that is serious, not studying it in pregnancy to me seems unethical… [A]nd on the other hand, just saying that you could study any drug in a pregnant woman, that doesn’t seem right either. So again, it depends on, to me, what the condition is, whether one studies it or not, and I would like to see more studies in pregnant women than less…instead [of being] reliant on observational gossip.” **Participant 328; researcher, region of the Americas**

While it was a minority view among experts, a few maintained that classifying pregnant women as a vulnerable population was warranted and extra precautions should be taken, even if these precautions slow development of new interventions for use during pregnancy.

“Pregnant women are [a] vulnerable group and just like [a] vulnerable group, they must be protected…. [W]hile we need more information for research, pregnancy is very sensitive and they must be protected, because certain damages done around that period may cause irreversible harm, so it’s a balancing act, whatever drives the research must be a balancing act.” **Participant 527; healthcare worker/researcher/ethicist, African region**

### Triangulation of Evidence for Evaluating Treatments in Pregnancy

Because of the lack of clinical trial data establishing efficacy and safety of interventions for use during pregnancy, all experts described the need for resourcefulness when evaluating new treatments for implementation with pregnant women. While they agreed that RCTs remain the gold standard for evaluating new clinical interventions, most recognized that obtaining RCT data for safety and efficacy during pregnancy was often not possible if there was any indication of fetal harm in preclinical trials. Registries were viewed as an essential but imperfect resource, given limitations on generalizability.

“[T]here will never be randomized controlled trial data on most of these things because anything that’s going to show up on an RCT would’ve had such a strong signal in preclinical trials that it would never have gotten there. … For me, the real question is, ‘How can you generate enough registry data to give yourself some confidence that you really believe it?’” **Participant 408; healthcare worker, region of the Americas**

In the absence of RCT data, all experts described triangulating and extrapolating data from animal studies, clinical case reports, and registry or post-market surveillance data to make decisions.

“[T]he more different sources of data we have, the easier it is to make decisions and to not feel like you are making decisions in the absence of information and that you can make informed decisions….” **Participant 131; researcher, African region**

However, many experts were quick to caution against several common errors in this approach. First was the need for careful consideration when extrapolating from currently available data to a different setting or population—important biological, genetic, and environmental differences could limit the meaningfulness of data from a particular population. Second, some experts noted that early reports of adverse outcomes, anecdotes, or case studies can be weighted heavily, and can stop interventions from moving forward to implementation research in pregnancy.

“I think many people are really influenced by anecdotal evidence and it usually just messes things up. I think it’s very important to have the real hard evidence in making these decisions, because RCTs would give us good evidence that would help us to make decisions based on some research being done properly.” **Participant 978; researcher, African region**

Other experts described how stories about single adverse events travel quickly and can breed mistrust among frontline clinicians and in the community, reducing uptake of potentially beneficial interventions, and reducing community members’ willingness to participate in research.

When asked about how best to weigh early data, clinical experts observed that policy makers can sometimes be overly cautious when early data suggests any risks during pregnancy, possibly granting too much weight to early animal data relative to the health impacts to women and their fetuses. One expert argued that even with some evidence of toxicity, this should be weighed against the benefits and use potentially limited to less vulnerable stages of later pregnancy, rather than taking an all-or-nothing approach:

“[T]he use of Efavirenz is another good example, where we had some animal data and some case reports suggesting issues with neural tube defects with use in the first few weeks of the pregnancy. [A]lthough that wasn’t definitive data, based on that, the FDA changed their recommendations. … In use later, we had no indication that there was an issue from the observational data…. So one could’ve said, ‘Well, you can’t use this drug in the first four weeks, but you can use it afterwards,’ but rather the FDA and the European group went way out and said, ‘You should never take this drug during pregnancy,’ which was an over-interpretation of the data.” **Participant 328; researcher, region of the Americas**

### A Nuanced Approach Needed When Balancing Maternal–Fetal Risk and Benefit

When we asked experts how they consider offering interventions to women during pregnancy, careful evaluation of the maternal/fetal risk–benefit ratio was central to their deliberation. Although this dilemma has often been framed as a dichotomous choice about whether to prioritize maternal or fetal interests, many experts resisted the dichotomy and argued that the woman’s and baby’s well-being are too intimately connected to be considered separately. Participants appealed to a range of ethical positions reflecting a continuum from a woman-centered prioritization to more infant-centered positions (Table 2).

**Table 2:** Positions on maternal–fetal prioritization and accompanying rationale

Position on maternal–fetal priority	Rationale offered	Example quotes
Prioritize woman over pregnancy	She can have another pregnancy/have another child but we can’t get another “her”	“I think the woman’s health should be a priority because she stands to get another pregnancy, so if she loses the one pregnancy she’s carrying or if she gets problems developing [the one] that she’s carrying, she’s still able to get another pregnancy. But, when we have a baby who can’t take care of themselves, the risks in the mother leads her to death, then we are enveloped in the hopeless situation of trying to raise this baby.” **Participant 691; healthcare worker, African region** “We know that if the mother loses the pregnancy, there is an opportunity to have another baby, but if we lose both the mother and the baby, that is a loss, so usually that is why we say that the mother is the priority.” **Participant 992; healthcare worker, African region** “As an obstetrician, I cannot delink the two…..as I take care of the health of the mother, the health of the baby is also important, but situations arise where [a] decision can be made in the interest of the health of the mother, but those are specific situations…..when you now know that is the only option you have, then you may make a decision in the interest of the mother that the mother will live for another day to have another baby. But those are really specific situations and cannot be generalized.” **Participant 527; healthcare worker/researcher/ethicist, African region**
Prioritize woman as patient	She’s the “living” patient	“A woman’s health takes priority. I mean you’re obviously going to consider both, but…….I mean she’s your patient at the outset. She’s the living object of your intervention until the baby’s born.” **Participant 586; healthcare worker/researcher, region of the Americas** “I think the mother’s health should be the priority, because we’re trying to protect the unborn baby not knowing whether it will be born alive or not…so I think the health of the mother should be the first priority in this case.” **Participant 995; researcher, African region** “[M]y experience from [country in sub-Saharan Africa] is that the fetus is not viewed as prominently as it is in [Western country], or it doesn’t seem to have as much importance…..because in countries like [country in sub-Sarahan Africa] so many infants die, that it’s much more accepted there than it is [in Western country], and I’m not saying that’s a good thing. But there’s also a greater tendency among obstetricians to give medications that we might not give in [Western country], knowing that there’s a risk to the fetus, because they don’t have other options. And so there is less attention paid to teratogenicity in countries like [country in sub-Saharan Africa].” **Participant 980; healthcare worker, region of the Americas**
Prioritize avoiding infant harm	Infant priority with serious risk of harm	“If we know that the risk to the infant is going to be severe, I think that absolutely matters. And if it’s [an] 80% chance that something’s going to happen to the infant, then there’s really no point rolling out that option, treatment or prevention.” **Participant 309; healthcare worker/researcher/policy developer, European region**
Mother first, because infant survival depends on her	Infant survival depends on the mother being alive	“And you know actually, the survival of children to a large extent is dependent on the survival of the mother, especially in our part of the world. And when you lose the mother, chances of losing the baby are also very high.” **Participant 202; healthcare worker/researcher/ethicist, African region** “….the ideal is to have a healthy mother in the future who can participate as much as possible in caring and raising the unborn child.” **Participant 741; healthcare worker/researcher, region of the Americas** “I would always go for the mother, protecting the life of the mother over the child, because of what we know and what evidence has shown about the risks to a child if the mother is unwell or the mother dies, then the child is at a higher risk, and especially in developing countries, so I would consider the health of the mother first and then the one of the infant.” **Participant 373; healthcare worker, African region**
Mother first, because family and community depend on her	She’s the mother of other children - think about her in terms of larger impact on community/family	“I think the health of the woman is absolutely paramount…. as much as there is concern regarding fetal exposure and fetal health, if we’re causing ill health to the woman, however small or big, I think that is something that really needs to be put into the balance, simply because she may be the mother of other children who need her, and she is a big contributor to the community and not only to her own family, and so it is absolutely paramount that the women’s health and women’s position be kept important in any decision, whether it’s research or in a clinical setting.” **Participant 309; healthcare worker/researcher/policy developer, European region** “If there is a definite benefit to the mother, then that is a treatment one needs to look at very favorably, because in the end, if the mother survives or if you are able to get out of danger, then not only is she able to look after any other children she has, but she can also get others. So, I mean, the benefits to the mother is the final consideration, and a very important one.” **Participant 202; healthcare worker/researcher/ethicist, African region**
Holistic	Healthy mom = healthy baby (in terms of treating an illness that might adversely affect the pregnancy)	“I feel like ultimately, even if I were to only think about the infant, it’s in the infant’s best interest to have a healthy mom. So I would say prioritize the maternal health because in fact a side effect of that is infant health.” **Participant 716; healthcare worker, region of the Americas** “I guess I’m always gonna land on what you would consider the side of the mother. But to me, the mother and the baby are sort of inseparable units, but if you’re treating the mother, it’s gonna benefit the baby in general.” **Participant 796; healthcare worker, region of the Americas** “[P]ersonally, I think [the health of] both is important and the mother’s health is critically important in order for ensuring that her infant reaches term and is delivered safely, so I don’t think her health should be excluded from the equation. But similarly, there should be a priority based on delivering a healthy infant and ensuring that there are no harms to that child because any harms to that child are likely to be life long and likely to be the responsibility of that women and her family and therefore, it’s important to bear that in mind as well.” **Participant 131; researcher, African region**

Among those who endorsed a woman-centered view, different reasons were offered. Some made an appeal to a woman’s right to make decisions over her own health. Others appealed to a personhood argument, describing the woman as “the patient” during the pregnancy, while they believed the fetus holds a lower moral status until term or delivery. On either account, these experts argued that we should minimize risks to the fetus, but prioritize the woman’s health over fetal health, especially when the mother’s health was at risk.

“I’m a maternalist, so I don’t see the life of the mother and the life of the fetus as equal. I think we need to put appropriate safeguards in place and do as little harm as possible to the fetus, but always keeping in mind that the mom’s life comes first. [G]enerally the way that I think about it is, if a mom really needs a drug for her health, then we need to treat her like she is not pregnant.” **Participant 980; healthcare worker, region of the Americas**

Other experts holding more woman-centered views along the spectrum strongly defended prioritizing the woman’s health on consequentialist grounds. These reasons included: (1) she can go on to have other children if she loses this pregnancy; (2) she has responsibilities to other children, her family, and her community; and (3) the infant’s survival and future well-being depend on the woman’s health.

However, within each of these ethical appeals, experts’ prioritization of maternal–fetal interests shifted, depending on the context. For example, clinical experts from WHO regions with high maternal and child morbidity/mortality were most inclined to collapse the dilemma of fetal vs. maternal interests, noting that the health and life prospects of the infant, and any other children the woman may have, depend on the health of the mother during and after childbirth. Others recognized that in many circumstances, including the case of HIV prevention and PrEP, the interests of both the woman and future baby are inextricably linked, requiring a more holistic consideration of potential benefits and risks, including that the woman will bear the care burden for any disabilities caused to the baby.

“[A]s a clinician, both are your patients and the infant’s health is tied to the mother’s health…..The mother’s health is critically important for ensuring that her infant reaches term and is delivered safely, so I don’t think her health should be excluded from the equation. But similarly, there should be a priority based on delivering a healthy infant and ensuring that there are no harms to that child because any harms to that child are likely to be life long and likely to be the responsibility of that woman and her family.” **Participant 131; researcher, African region**

When asked how the interests of the woman and her unborn baby ought to be balanced in the context of HIV prevention, and more generally in the provision of any intervention during pregnancy, experts considered the severity of the illness in the woman against the likelihood of fetal harm as the main consideration. When the illness was severe and the mother’s life was in jeopardy, these experts prioritized intervention regardless of the impact to fetal health.

“The maternal health piece, in almost all cases, would be the primary consideration; you would want to be sure that you are treating a woman for a serious condition adequately, and unless you knew that the agent being used had serious consequences for the fetus, the maternal benefit would outweigh it, and potentially, if you had a life-threatening illness in the mother, even if you knew that there might be a problem in the fetus, one might consider moving forward, if you were saving that woman’s life.” **Participant 328; researcher, region of the Americas**

When evaluating outcomes, the majority of experts placed more weight on known harms to the mother or fetus when compared to potential fetal harms. In certain situations, known fetal harms trumped potential benefits to the woman.

“[T]he sicker the woman and the more provenly beneficial the treatments are for the woman’s sickness, the more likely you are to accept the potential for known or unknown risk to the fetus.” **Participant 408; healthcare worker, region of the Americas**

Some experts thought it always ethically required to minimize the use of treatments with potential or unknown risks to the fetus, except when there is no alternative for the mother, and this position was often accompanied by reflection on past controversies. One participant appealed to the future child’s right to as open and healthy future as possible.

“Well, you know there’s the old Thalidomide story, right? That the potential for harm and the lives of those people born with the harmful effects, I think they too have the right to the potential of a healthy future life, so the issue of protecting the unborn fetus so that when they’re born, they’re born as healthy as is possible, is important.” **Participant 660; researcher, African region**

### Considering Economic Factors, Logistical Considerations, and Cultural Attitudes About Women’s Autonomy in Local Contexts

Experts shared many insights on the contextual factors influencing decisions to move forward with new interventions for use in pregnancy (Fig. 1).

“I think the social and economic context of the clinical care and research is always part of the equation, too. [W]omen in some places are desperate for good medical care, and some places they’re not. And I think when we talk about how we’re going to take care of women when they’re pregnant… we can’t divorce ourselves from that economic and social context, which affects every decision that they make, basically.” **Participant 782; researcher/ethicist, region of the Americas**

Within resource-constrained settings, medication costs play a large role in treatment decisions during pregnancy. Cost and limited financial resources can impact the availability and acceptability of treatment options when women or in-country policy makers are asked to balance medication costs with treatment effectiveness and the availability of alternative treatment options.

“PrEP has great potential [for] preventing infection. But we have so many people who need treatment and I think sometimes I understand the dilemma of the policy maker because with the resources they have, who do they give [PrEP to], do they give to the sick or do they use the resources to prevent new people from getting [sick]…? We don’t have the unlimited resources.” **Participant 202; healthcare worker/researcher/ethicist, African region**

In addition to cost, logistical factors, such as dosing, availability of alternative options, and pick-up location were important practical factors experts considered when making treatment decisions. Medications requiring too much time, follow-up, or intensive care were recognized as not likely to be taken up by women and therefore less likely to be prescribed.

“[W]ithin the environments where we work you’ve got overloaded services already. We battle to get women into four antenatal visits. We battle to get HIV-positive women into three monthly visits for testing, and negative [women] for testing…Anything that’s just too demanding is going to be highly challenging, unless it’s just a very small number of women that require that level of intensity to treat them, like diabetics…And I know it’s a consideration for the woman because if she’s having to take time off work, pay for fare, spend a day sitting in the clinic, you know she’s not going to be keen to do that either.” **Participant 539; researcher, African region**

All experts described the challenges of navigating social and cultural attitudes towards the role of women in decision-making, including attitudes of deference from women themselves. In situations of uncertainty, when information or data are imperfect, experts still believed it is important to share what is known with women. This was especially true in situations where the decision is complex and both the mother’s health and the health of the fetus are in jeopardy. Clinicians, in particular, felt it was important to make such a decision with a woman as a shared decision, to support women in weighing the risks and benefits as known, together. Overall, the more uncertainty present in the risk/benefit equation, the more experts wanted the woman to be informed and make her own choice, but with support. Clinical experts reported that women differ in their desire for information and involvement in decisions and found it challenging when women defer to their expertise, but they do not have sufficient evidence on which to base a recommendation.

“Some women want to be very involved with every decision-making, and there are some women who simply want a clinician to tell them what the clinician thinks is best. And that’s tricky when it’s a grey area and the clinician can’t say definitely what’s best.” **Participant 221; healthcare worker, region of the Americas**

Clinical experts also found it difficult to navigate conversations with pregnant women who prioritized the safety of the fetus at the expense of their own health, with some noting that they would feel obligated to advocate for the woman’s health in such a case.

“[M]ost of them in my experience, whether it’s in Europe or in Africa, most women will have a bias towards the baby’s health…but I think it’s up to us to help make sure that we balance that a little bit. If something is really dangerous for the health of the woman…we should really advise them towards their own health.” **Participant 136; healthcare worker/researcher/ethicist, European region**

Similarly, clinical experts described the cultural challenges surrounding women’s deference to male partners and expectations in some cultural contexts that male partners must be involved in decisions potentially affecting a woman’s pregnancy. Several experts took a pragmatic approach in considering male partners’ views in contexts where they knew women were likely to defer to husbands.

“The male partner becomes an important player and stakeholder, and quite often even after you have discussed issues with the women, they defer, they don’t make a decision, they want to go and consult first, so that becomes important because whatever he decides is probably what is going to carry the day.” **Participant 202; healthcare worker/researcher/ethicist, African region**

Finally, several clinical experts made an important observation about the role of women in making decisions around new interventions for use in pregnancy, noting that particularly in developing countries, women are seldom brought into the decision-making processes about when and what new drugs and interventions are made available to pregnant women in the first place.

“I’ve heard this in these conversations where people are talking about rolling out PrEP… particularly in the developing world… where there’s this very patriarchal way of thinking: there are bodies of people who are going to make decisions about what’s best for women, and that kind of rubs me the wrong way. I still think that there has to be this theme through what we do about giving them the information and then helping them in making the choice that works best for them. **Participant 221; healthcare worker, region of the Americas**

## Discussion

This study provides important insight into how clinical and policy experts in HIV prevention wrestle with the implementation of new HIV prevention interventions for pregnant women, or for women who may become pregnant in low-income countries with a high HIV-burden. The recent history and resulting culture of HIV research has likely been influenced by more than two decades of research with pregnant women on PMTCT, in a sense, making inclusion of pregnant women the rule rather than the exception. Against this backdrop, the exclusion of pregnant women from clinical trials of PrEP is striking. The timing of the debate around whether to implement PrEP during pregnancy offered a unique opportunity to understand how experts weigh early and often incomplete evidence and ethical considerations for an intervention aimed primarily at preventing HIV in the woman. The findings offer a valuable glimpse into the complicated, contextual deliberations of experts that have informed or will inform clinical guidelines and policies for introducing a new intervention aimed at preventing a woman’s acquisition of HIV during pregnancy and lactation.

Our interviews with clinical and scientific experts working in HIV and maternal-child health reveal that providing evidence-based interventions for women during pregnancy is influenced by complex and often implicit scientific, ethical, practical, and socio-cultural judgments. There is both ethical and clinical value in making such judgments explicit to reflect on how to improve deliberations when moving from research to implementation of safe and effective interventions for use by pregnant women. As our group of experts acknowledged, decisions about which new drugs and interventions ought to move forward to implementation studies to evaluate use in pregnancy are often made by panels of experts with highly imperfect information, making it important to understand how experts are weighing imperfect sources of evidence against ethical, cultural, and practical considerations.

While many in international research ethics and policy have strongly argued against the categorization of pregnant women as a vulnerable population, and the recent Council for International Organizations of Medical Sciences (CIOMS) guidelines reflect this position [1], US regulations and many other country-level guidelines governing human subjects protection still classify pregnant women, together with fetuses and neonates, as vulnerable populations. The impact of limited research in pregnancy on the availability of efficacious treatment options is well recognized. Our case study of PrEP supports the need for more evidence of drug safety during pregnancy, and improved mechanisms for triangulating and sharing existing data in a systematic way. Findings also suggest a need for development of decision-making tools to help policy makers balance the risks and benefits of existing evidence in more nuanced ways, considering maternal–fetal risks, and benefits, within socio-economic context.

Women make up the majority of new HIV infections in African countries, and young women of reproductive age are at even greater risk [12–14]. Yet, pregnant and lactating women were excluded from clinical trials of prophylaxis for HIV due to concerns of unknown risks to the developing fetus. The experts we spoke to described the paradox of underinclusion in clinical trials: lack of evidence justifies exclusion of pregnant women when erring on the side of caution; but exclusion simply delays the need for evidence. For example, as we have seen in the case of PrEP, while international guidance from WHO has recommended use of PrEP in pregnant women, there is confusion and inconsistency at the country level. Some countries have followed international recommendations and other countries, such as South Africa, are currently not recommending use in pregnancy, listing pregnancy and breastfeeding as contraindications to PrEP despite agreeing that PrEP “should be tailored to populations at highest risk of HIV acquisition [18, 24].”

Underinclusion also creates confusion at the clinical bedside. Experts in our study argued that lack of available evidence-based prevention and treatment options for pregnant and lactating women simply shifts two significant ethical dilemmas to practicing clinicians: (1) it creates an unequal distribution of risks and benefits on pregnant patients compared to non-pregnant patients and (2) it puts the clinician in the position of having to balance maternal and fetal risks and benefits to best protect the health and welfare of both parties, but without sufficient information to offer women. This is acknowledged as a serious gap at the country level, again considering the example of South Africa’s policy guidance on PrEP, which states, “The use of PrEP around the time of conception and during pregnancy offers a means of protection to the uninfected partner. Unfortunately, data relating to the safety of PrEP specifically with regard to the developing foetus are limited, and consequently the onus is on the clinician to discuss potential risks and benefits of PrEP initiation or maintenance during pregnancy with the client [24].”

Experts participating in our study recognized the importance of research during pregnancy and wrestled with how to make clinical and policy judgements based on currently available evidence. This challenge of decision-making under uncertainty is not unique to PrEP. Immunization against infectious diseases and TB medication use during pregnancy are also plagued with a scarcity of data to inform decisions [25]. Although RCT data is preferred, experts acknowledged the serious challenges involved in responsibly gathering “gold standard” data without exposing pregnant women to medication that is potentially harmful to the developing fetus. Many expressed deep concern about the possibility of “another Thalidomide”, while simultaneously pointing to the fact that had a trial of Thalidomide in pregnant women been conducted, fewer fetuses would have been harmed.

Given the paucity of RCTs, experts described resourceful strategies for considering alternative sources of data including animal studies, data from other populations and registries. While clinical experts in our study found this information valuable and used it to make clinical decisions, they also noted a need for more robust data-gathering strategies and described ways of triangulating data sources. One barrier to systematically gathering early evidence for safety and efficacy in pregnancy is the potential chilling effect of anecdotes, or a single patient case study involving adverse outcomes, to shut down the careful path of inquiry into new interventions for pregnant women. This observation reinforces similar insights from bioethicists who have argued that, while other areas of medicine may allow more time and space for carefully building the evidence during periods of uncertainty, pregnancy research remains much more conservative and reactive about possible risks [5].

One of the most interesting findings from the study was the subtlety of reasoning about an often oversimplified ethical tension in maternal–fetal risk assessment. Overall, experts in our study evaluated complex considerations, including available evidence, maternal/fetal risk–benefit analysis, and the social/cultural context in which the decision is being made. Within the maternal–fetal risk analysis, experts largely resisted the dichotomy between putting a woman’s health first versus preventing harm to the fetus, rarely allowing one to trump the other. Instead, even those who clearly prioritized the woman’s health offered nuanced thinking about obligations to minimize harm to the developing fetus, including consideration of a woman’s own deep concerns about preventing harm to her baby. Most in our group argued for the inextricably linked interests of woman and developing fetus and appealed to context to strike an optimal balance. Experts from resource-limited countries, especially, took a more holistic view of the woman and fetus, observing that not only the future baby, but other children in the household and the wider family will depend on the woman for survival. For this reason, in their view, protection of the woman’s health is necessary for ensuring infant survival.

While half of the experts prioritized maternal health over fetal health, the rest felt that maternal and fetal interests were intimately connected and that the forced dichotomy of maternal versus fetal health prioritization was not an accurate representation of their decision-making process. This has important implications for how we think about the ethics of maternal–fetal conflicts in research. When evaluating mixed or incomplete evidence, experts weighed two factors more heavily in decisions to offer a new medication for use in pregnancy: (1) the severity of the potential disease outcomes for the woman, and (2) the availability of alternative interventions that are known to be safe for a developing fetus. More fetal risk was considered acceptable for life-threatening or very severe illnesses, and potential fetal exposure to harmful medications was acceptable when lack of treatment would have serious consequences for the women’s health. In addition, if alternative treatment options were available, experts were willing to trade maximal efficacy in the woman for safety in the fetus, such that a moderately effective drug with a good safety profile rated higher than a very effective, but potentially risky, medication. Findings suggest the need for a more systematic study of ethical deliberation under uncertainty regarding treatment decisions in pregnancy and development of tools to support such decision-making. This would offer an important next step for better understanding the underlying ethical rationale behind priority setting when considering maternal and fetal interests and offer greater clarity during the challenging transition period from research to clinical practice for new interventions for pregnant women.

There are a number of limitations to the study. While the information experts provided is valuable for understanding general decision-making practices, participants were not asked to respond to actual data about specific interventions, or their experiences actually providing PrEP in practice to pregnant women, during the interview. Instead, experts relied on recollection and professional expertise regarding the current literature on HIV prevention interventions and PrEP, which likely varied across participants, as did personal clinical experience versus policy experience. However, soliciting decision-making rationale and better understanding value judgements about treatment during pregnancy can inform how policy makers and clinicians evaluate the use of new medications in pregnant women in the future. As PrEP access continues to expand, exploring actual experiences providing PrEP to pregnant women should be explored. Given the inherent interest in how experts reason through these important decisions, we do not include our data on women’s, frontline clinicians’, and partners’ views here. These are equally important perspectives in this debate and, as analysed, will be available separately, with comparative reference to this cohort [26–28].

## Conclusion

The global HIV epidemic and the experience with mass inclusion of pregnant women in PMTCT research offers an interesting historical lesson and opportunity to reflect on the importance of including pregnant women in research. The continued high global burden of HIV in women and susceptibility of women to HIV during pregnancy, underscore the need for interventions to prevent HIV acquisition during pregnancy and support reconsideration of policies for including pregnant women in research [2, 3]. Without randomized trial evidence for PrEP use in pregnancy, policy-makers and clinicians triangulated evidence to support use and implementation of PrEP in pregnancy. Our analysis of clinical and policy expert considerations regarding use of PrEP in pregnant women demonstrates how thoughtful deliberation under both clinical and ethical uncertainty can nonetheless increase access to effective safe treatments for pregnant women. Our findings suggest that experts and clinicians support wider inclusion of pregnant women in randomized trials that evaluate both maternal and infant outcomes. If inclusion of pregnant women is not possible for an interventional RCT, early identification of relevant or alternative sources of data that could accelerate safe effective treatment access for pregnant women is important. Further research on the types and levels of evidence that persuade clinicians and experts that an intervention is safe and effective in pregnancy could inform a more systematic pathway towards intervention access for pregnant women.

## Electronic supplementary material

Below is the link to the electronic supplementary material.

Supplementary material 1 (DOCX 15 kb)
