# Supplementary material for: Lessons from PrEP: A Qualitative Study Investigating How Clinical and Policy Experts Weigh Ethics and Evidence When Evaluating Preventive Medications for Use in Pregnant and Breastfeeding Women
Source: AIDS Behav. 2018 Dec 14;23(7):1858–70. doi: 10.1007/s10461-018-2361-5 (PMC6570782; doi:10.1007/s10461-018-2361-5)
Supplement: Supplementary file 1 — Supplementary material 1 (DOCX 15 kb) [file 10461_2018_2361_MOESM1_ESM.docx]

**Supplemental Material:** Interview Guide

**I. Clinical Treatment (General)**

I want to start by asking about the challenges that may arise in the **clinical treatment** of women who are pregnant or breastfeeding, specifically in the case when a medication can help the woman but has unknown effects on the developing infant.

1. In general, do you think clinicians should offer a particular medication to pregnant or breastfeeding women in this situation?
   1. Why or why not?
2. How do you think the woman’s health should be weighed in decision making relative to the potential risk to the fetus/infant?
3. Next I’d like learn about what context-specific factors might inform your thinking about whether a medication should be offered to patients who are pregnant or breastfeeding, when effects on the fetus are uncertain or unknown. For example, to what degree would it matter whether:
   1. Whether the intervention is to prevent or to treat a condition?
   2. Whether the treatment/prevention is for a sexually transmitted infection vs. other disease?
   3. Now I’d like to ask about two different components of risk to the fetus/infant:
      - First, how **severe** the potential risk is for infant?
      - Second, how **likely** the potential risk is for infant?
   4. What type of evidence on safety or harm is available? – e.g., RCT vs. anecdotal reports?
   5. What type of evidence is available on the effectiveness of the medication? – e.g., 95% effective vs. 25% effective
   6. Whether the intervention is considered part of routine care for non-pregnant women?
   7. The woman’s/mother’s preferences?
   8. The partner’s preferences?
   9. Practical/logistical considerations – e.g., retention, ease of follow-up, access to medications, dosing regimen (1/day vs. 1/month)
   10. Are there any other factors that might influence your thinking?
4. I asked about the woman’ preferences briefly, but now I’d like you to say a bit more about women’s views and decision-making.
   1. In your opinion, how much choice do you think women want to have over what medications they take during pregnancy?
   2. Slightly different than what they want, what do you think their role in medical decision-making should be? How much discretion should women be given over medical decisions for their own health that may impact their pregnancy/infant?
   3. Do you think about this differently when we’re talking about using new interventions during pregnancy? – [for clinician: do you share what is known or not known about new interventions, in terms of safety/efficacy during pregnancy?]

**If the expert is a clinician:**

- 1. What do you do or say if the woman’s opinion or decision is different from yours?
  2. What do you do when the patient’s preferences are different from her partner’s wishes?

1. How do you think clinicians should approach informed consent with pregnant or breastfeeding patients when effects on the fetus are uncertain or unknown?
2. Is there a particular NGO or professional society you would look to for guidance on this question?

**II. Intervention Research (General)**

Thank you. Now I’d like to ask about your views with respect to **intervention research** with women who are pregnant or breastfeeding.

Under current regulations and guidelines, the scope of allowable research with pregnant women is quite limited, due to concern for the welfare of the fetus/infant. Some experts believe that this caution is justified, and research ought to be limited. Others feel that more research ought to be done, with appropriate protections and oversight to minimize the risk to developing fetuses/infants.

1. What are your thoughts on this policy question in human subjects protection? *[Probe, as needed, to learn the underlying values/rationale for the participant’s opinion.]*
2. Do you feel that the community standard of care should be taken into consideration in deciding what research is allowable?
3. How much should the local/regional burden of disease inform policy on the inclusion of pregnant women in research?
4. As in the previous section, are there contextual factors that would make a difference in your thinking about the question of which research should be done?
   1. Whether the intervention is to prevent or to treat a condition?
   2. Whether the treatment/prevention is for a sexually transmitted infection vs. other disease?
   3. Now I’d like to ask about two different components of risk to the fetus/infant:
      - First, how **severe** the potential risk is for infant?
      - Second, how **likely** the potential risk is for infant?
   4. What type of evidence on safety or harm is available? – e.g., RCT vs. anecdotal reports?
   5. What type of evidence is available on the effectiveness of the medication? – e.g., 95% effective vs. 25% effective
   6. Whether the intervention is considered part of routine care for non-pregnant women?
   7. The woman’s/mother’s preferences?
   8. The partner’s preferences?
   9. Practical/logistical considerations – e.g., retention, ease of follow-up, access to medications, dosing regimen (1/day vs. 1/month)
   10. Are there any other factors that might influence your thinking?
5. Is there a particular NGO or professional organization you would look to for guidance on this question about the inclusion of pregnant women in research?
   1. What additional guidance would you find helpful?

**III. Implementation of PrEP**

Thank you. For this final section, we will shift to a more specific intervention. Some experts feel that it is time to begin to offer pre-exposure prophylaxis (PrEP) to pregnant and breastfeeding women for the prevention of HIV. Others feel that more evidence of efficacy in women and safety in fetuses/infants is needed before widespread implementation takes place.

1. What are your thoughts on this issue? Is PrEP ready for clinical use in pregnant women?
   1. Why or why not?
2. Is there a difference, in your mind, between PrEP use in pregnant women vs. breastfeeding women? Any different concerns or thresholds?
3. Can you think of any specific barriers to the implementation of PrEP in pregnant and breastfeeding women in low-resource settings?
4. How would you compare PrEP for HIV prevention with the examples of malaria and TB prophylaxis?
   1. Is PrEP different?
   2. Why or why not?
5. In your experience, why do some preventive interventions move forward and others not?
6. Can you think of any key individuals or groups that would have a significant influence on whether PrEP is more broadly implemented to prevent HIV in pregnant women?
7. Is there anything else you think we should know about this issue?

We know you are very busy. Thank you so much for sharing your time and expertise. We really appreciate it. Would you be interested in learning about our findings at the end of the study?

Can you think of anyone else you think we should be talking with? Again, thank you.
